# Embryonic Stem Cell-Derived Neurons Grown on Multi-Electrode Arrays as a Novel *In vitro* Bioassay for the Detection of *Clostridium botulinum* Neurotoxins

**DOI:** 10.3389/fphar.2017.00073

**Published:** 2017-02-23

**Authors:** Stephen P. Jenkinson, Denis Grandgirard, Martina Heidemann, Anne Tscherter, Marc-André Avondet, Stephen L. Leib

**Affiliations:** ^1^Neuroinfection Laboratory, Institute for Infectious Diseases, University of BernBern, Switzerland; ^2^Biology Division, Spiez Laboratory, Swiss Federal Office for Civil ProtectionSpiez, Switzerland; ^3^Cluster for Regenerative Neuroscience, Department for Clinical Research, University of BernBern, Switzerland; ^4^Graduate School for Cellular and Biomedical Sciences, University of BernBern, Switzerland; ^5^Department of Physiology, University of BernBern, Switzerland

**Keywords:** botulinum neurotoxins, BoNT, botulism, embryonic stem cell-derived neurons, neuronal network, *in vitro* bioassay, multi-electrode array, MEA

## Abstract

*Clostridium botulinum* neurotoxins (BoNTs) are the most poisonous naturally occurring protein toxins known to mankind and are the causative agents of the severe and potentially life-threatening disease botulism. They are also known for their application as cosmetics and as unique bio-pharmaceuticals to treat an increasing number of neurological and non-neurological disorders. Currently, the potency of biologically active BoNT for therapeutic use is mainly monitored by the murine LD_50_-assay, an ethically disputable test causing suffering and death of a considerable number of mice. The aim of this study was to establish an *in vitro* assay as an alternative to the widely used *in vivo* mouse bioassay. We report a novel BoNT detection assay using mouse embryonic stem cell-derived neurons (mESN) cultured on multi-electrode arrays (MEAs). After 21 days in culture, the mESN formed a neuronal network showing spontaneous bursting activity based on functional synapses and express the necessary target proteins for BoNTs. Treating cultures for 6 h with 16.6 pM of BoNT serotype A and incubation with 1.66 pM BoNT/A or 33 Units/ml of Botox^®^ for 24 h lead to a significant reduction of both spontaneous network bursts and average spike rate. This data suggests that mESN cultured on MEAs pose a novel, biologically relevant model that can be used to detect and quantify functional BoNT effects, thus accelerating BoNT research while decreasing animal use.

## Introduction

The BoNTs are produced and secreted by the bacteria *Clostridium botulinum, C. butyricum*, and *C. baratii* and are amongst the most lethal substances known, with an estimated human lethal dose of 0.1–2 ng/kg if applied intravenously (Arnon et al., 2001; Simpson, 2004). Seven BoNT serotypes (A–G) and more than 40 subtypes are currently described, where BoNT/A, B, E, and F have been directly associated with human illness causing symptoms of botulism with four naturally occurring syndromes (Simpson, 2004; Lindstrom and Korkeala, 2006; Rusnak and Smith, 2009). Recently, the California Department of Public Health published data on the finding of a *C. botulinum* strain IBCA10-7060 that produced BoNT/B and another BoNT that could not be neutralized by any of the provided monovalent polyclonal botulinum antitoxins raised against BoNTs A–G (Barash and Arnon, 2014). The novel BoNT was thereupon described as a newly defined serotype H. However, this has been questioned by several groups and additional studies were recommended to confirm its identity (Johnson, 2014; Rossetto et al., 2014). Recent published data has now shown that BoNT/H (alternatively termed BoNT/FA) has a chimeric structure containing regions similar to the structures of BoNT/A1, BoNT/F1, and BoNT/F5 (Kalb et al., 2015; Maslanka et al., 2016; Pellett et al., 2016).

Foodborne botulism is caused by ingestion of BoNT contaminated food whereas wound botulism is caused by germination of *C. botulinum* spores in wounds and *in situ* toxin production. Infant botulism as well as the rarest form, the adult botulism, results through ingestion of spores which can germinate in the intestinal tract and produce BoNTs (Lindstrom and Korkeala, 2006). Clinical symptoms occur 2–36 h after exposure, depending on dose and route of admission and are characterized by a generalized weakness which progresses to a flaccid paralysis that can ultimately lead to respiratory arrest and subsequent death due to suffocation (Bohnel and Gessler, 2005).

All BoNTs are synthesized as 150 kDa polypeptides and processed by post-translational proteolytic cleavage to yield a 100 kDa HC and a 50 kDa LC linked by a disulfide bond. They are further subdivided in functionally independent domains. Neuronal cell entry of the toxin is mediated by a multi-step process requiring fully functional BoNT holotoxins (Brunger and Rummel, 2009). In a first step, the H_C_ domain, located at the C-terminal portion of the HC, mediates binding to polysialo gangliosides (e.g., GD1a and GT1b) which are present in high density on the presynaptic membrane, thus establishing the initial anchorage to the neuronal membrane (Rummel, 2016). Upon successful binding toward these polysialo gangliosides, BoNTs bind additionally via the H_C_ domain to different synaptic vesicle receptors located on the presynaptic membrane. Specifically, BoNT/A, D, E and F bind to the SV2 receptor and BoNT/B and G bind to synaptotagmin I/II (Rummel, 2016). After binding to both receptors, BoNTs are internalized by receptor mediated endocytosis (Montal, 2010). Upon successful uptake, the H_N_ domain, located at the C-terminal portion of the HC, translocates the LC into the neuronal cytosol where it exerts its enzymatic activity, acting as a zinc-dependent endopeptidase (Fischer and Montal, 2007). LC specifically targets and cleaves different SNAREs which play crucial roles in synaptic exocytosis (Schiavo et al., 2000; Montal, 2010). In particular, BoNT/A, C and E cleave SNAP-25 and in addition BoNT/C also cleaves syntaxin whereas BoNT/B, D, F, and G cleave the vesicle associated membrane protein-1 and -2 (Binz, 2013; Rossetto et al., 2014). Proteolysis of any of these three proteins prevents the assembly of the conserved synaptic exocytosis complex, thus blocking neurotransmitter release leading to the symptoms associated with clinical botulism.

BoNT/A and to a lesser extent BoNT/B are used as pharmaceuticals to treat a variety of neuromuscular disorders, chronic pain and in cosmetics (Jankovic, 2004; Naumann et al., 2008). Due to their extreme high potency, these pharmaceuticals need to be carefully titrated to ensure clinical benefits with minimal side effects. Furthermore, BoNT has been classified as a category A Select Agent due to the high lethality, lack of therapeutic options and potential misuse as a bioterrorism weapon (Arnon et al., 2001; Bossi et al., 2006).

Currently, the standard method for BoNT detection and quantification makes use of an *in vivo* mouse bioassay (MBA), in which the test material is injected intraperitoneally or intravenously in mice and signs of toxicity are observed until death occurs due to respiratory arrest (Schantz and Johnson, 1990). The MBA is a sensitive and robust assay, but faces several disadvantages: a large number of animals are required, lab to lab variations can occur, high costs accrue and up to 4 days are necessary to yield results. The assay also inflicts severe suffering to the mice and may lead to suffocation within the test period, underlining the need for alternative *in vitro* methods able to reduce or replace the use of live animals (Adler et al., 2010). Many different *in vitro* assays have already been established, which are capable of detecting BoNTs or BoNT catalytic activity with an equal or higher sensitivity than the MBA. Depending on the type of samples to be analyzed (highly purified toxin or crude samples), different prerequisites for the assay are necessary. For the analysis of reported botulism cases in humans and animals, the assay must detect all serotypes in a fast manner and has to be compatible with different environmental, clinical, and food matrices (e.g., serum, feces). In contrast to the pharmaceutical product, those samples require only a rough estimate of the toxin’s catalytic activity. Established and sensitive methods capable of detecting BoNTs in complex matrices include immunological detection methods (e.g., direct or indirect ELISA) or endopeptidase assays (Dorner et al., 2013). For the analysis and potency testing of pharmaceutical preparations the assay must determine the toxin’s catalytic activity in a highly precise manner and has to detect all steps of BoNT action, including binding to specific receptors, internalization into neurons, translocation of the LC into the cytosol and proteolytic cleavage of SNARE proteins. Duration of the assays and matrix effects are not relevant to the testing of pharmaceutical products, since they are composed of highly pure toxins or toxin complexes diluted in physiological buffer containing additives and stabilizing proteins. ELISA assays do not differentiate between holotoxin and reduced toxin in which the HC and LC are separated and endopeptidase assays only detect the proteolytic activity of the LCs, thus hold several important restrictions in replacing the MBA for the analysis and potency testing of pharmaceutical preparations (Pellett, 2013).

In contrast, neuronal cell-based assays provide a model which includes all steps of cellular intoxication, including binding to the cell surface, endocytosis, translocation of the LC into the cytosol and enzymatic activity of the LC toward SNARE substrates. Several cell-based assays using continuous cell lines, primary neuron cultures or stem cell-derived neurons have been developed over the past years exceeding the sensitivity of the MBA thus offering an alternative method for BoNT potency determination (Pellett, 2013). In fact, Allergan Inc., the distributor of Botox^®^, published data for an alternative detection assay using a continuous cell line which has been approved by the Food and Drug Administration as a replacement method for potency testing of pharmaceuticals (Fernandez-Salas et al., 2012). This assay, along with others, uses SNARE cleavage as endpoint readout for BoNT activity which can be determined in cell lysates by Western blot or by direct/indirect ELISA. Therefore, they depend on destructive homogenization of tissue to allow quantification and assessment thus requiring additional methods and further hands-on time to yield a result (Nuss et al., 2010; Pellett et al., 2010, 2011; McNutt et al., 2011; Whitemarsh et al., 2012). SNARE cleavage has also been determined in live cells by quantitative immunofluorescence using cleavage-specific antibodies enabling a potential high-throughput method for the detection of BoNT/A (Kiris et al., 2011). By using FRET and introducing a SNARE-FRET construct transiently into PC12 cells, that undergoes a change in fluorescence emission upon successful BoNT-mediated cleavage, Dong et al. (2004) were able to detect the biological activity of BoNT/A. An alternative endpoint readout for the analysis of BoNT activity is the inhibition of neurotransmitter release that can be determined by different approaches, e.g., pre-loading cells with radioactively labeled neurotransmitter (Keller and Neale, 2001; Sheridan et al., 2005) or endogenous neurotransmitter release can be directly measured by high-performance liquid chromatography or immunoassays (Welch et al., 2000; McNutt et al., 2011). Another approach is whole-cell patch-clamp recordings of mESN to evaluate changes in synaptic transmission following treatment with BoNT/A. This synaptic function assay revealed to be more sensitive than molecular readouts of SNARE substrate cleavage (Beske et al., 2015).

Multi-electrode arrays represent a unique tool to investigate network dynamics and allow a concurrent and non-invasive recording of electrical activity from many neurons and other cell types simultaneously. In the past, these have been widely used to characterize the spontaneous and evoked activity of neuronal networks (Shahaf and Marom, 2001; Tscherter et al., 2001; Bonifazi et al., 2005; Ban et al., 2007). Of note, the effects of neuroactive compounds on neurons cultivated on MEAs can be easily monitored (Pancrazio et al., 2003, 2014; Johnstone et al., 2010; McConnell et al., 2012).

In the present study, we have established a novel *in vitro* method where alterations in synaptic transmission after BoNT/A treatment are detectable in mESN grown on MEAs. We could show for the first time that these neurons are susceptible to BoNT/A treatment and that the assay was able to detect a dose- and time-dependent inhibition of synaptic transmission to picomolar (pM) concentrations of BoNT/A and the commercially available pharmaceutical BOTOX^®^.

## Materials and Methods

### Mouse Embryonic Stem Cell Culture

Wild type mESCs (E14) (Hooper et al., 1987), a kind gift of Marta Roccio (Inner ear research lab, Department of Clinical Research, University of Bern, Switzerland) were maintained in a 37°C tissue culture incubator at 5% CO_2_ and differentiated into mESN using previously described protocols with several adaptations (Wichterle et al., 2002; Wichterle and Peljto, 2008; Wu et al., 2011). Briefly, cells were maintained in tissue culture flasks (TPP, Trasadingen, Switzerland) that had been coated for 30 min at 37°C with 0.1% gelatine in ES medium consisting of DMEM with Glutamax, 15% fetal bovine serum, embryonic stem cell-qualified (GIBCO^®^, Thermo Fisher Scientific, Waltham, MA, USA) supplemented with MEM non-essential amino acids to a final concentration of 1 mM for all amino acids (glycine, L-alanine, L-asparagine, L-aspartic acid, L-glutamic acid, L-proline, L-serine), penicillin-streptomycin-glutamine to a final concentration of 1000 Units/ml penicillin, 1000 μg/ml streptomycin, and 0.292 mg/ml L-glutamine, 2-mercaptoethanol (0.1 mM) as well as leukemia inhibitory factor to a final concentration of 1000 Units/ml (Merck Millipore, Darmstadt, Germany). Unless otherwise specified, all cell culture supplies for embryonic stem cell maintenance, mESN differentiation and mESN culture were obtained from Invitrogen (Paisley, Scotland, UK). To elicit embryoid body (EB) formation and induce differentiation of the mESCs, 1–2 × 10^6^ cells were plated on day 0 in a 10 cm tissue culture dish (TPP, Trasadingen, Switzerland) and the medium was changed to a differentiation medium termed DMNK^+^ consisting of a 1:1 mixture of DMEM-F12/Glutamax and Neurobasal medium, supplemented with penicillin-streptomycin-glutamine to a final concentration of 1000 Units/ml penicillin, 1000 μg/ml streptomycin, and 0.292 mg/ml L-glutamine, 2-mercaptoethanol (0.1 mM), and 15% KnockOut^TM^ Serum Replacement. After 1 day in culture the floating EBs were transferred into a 15 ml tube, centrifuged at low speed (3 min; 35 × *g*), resuspended in 10 ml of DMNK^+^ medium and replated in a new 10 cm dish. On day 2 of differentiation, EBs were supplemented with 1 μM retinoic acid to induce neuralization. The next day, the medium was supplemented with 1 μM of smoothened agonist (Merck Millipore, Darmstadt, Germany). On day 7, EBs were dissociated using 2 ml of Accumax (Merck Millipore, Darmstadt, Germany) for 10 min at room temperature, diluted with 2 ml of DMNK^+^ medium and carefully pipetted up and down 30 times using a 1000 μl pipette. Cells were filtered through a 100 μm cell strainer (Becton Dickinson, Bedford, MA, USA) to obtain a single cell suspension. This step was performed three times to enrich the yield of single cells. The suspension was then centrifuged (3 min; 35 × *g*) and resuspended in 2 ml of DMNK^+^ medium supplemented with recombinant rat glial cell-derived neurotrophic factor (10 ng/ml), recombinant human brain-derived neurotrophic factor (10 ng/ml), and recombinant rat ciliary neurotrophic factor (20 ng/ml) (all R&D Systems, Minneapolis, MN, USA). 2 × 10^5^ cells/ml in 100 μl of DMNK^+^ medium supplemented with factors were then plated on MEAs which were previously coated for 30 min at 37°C with Matrigel^TM^ (Corning, Corning, NY, USA) diluted in 1:10 DMNK^+^ medium. The cells were then cultured at 37°C, 5% CO_2_ changing medium every 3 days. Exposure to toxins and electrophysiological recordings were conducted 21 days (±2 days) after plating.

### Multi-Electrode Arrays

Multi-electrode array slides (Qwane Biosciences, Lausanne, Switzerland) composed of a glass substrate (700 μm thick, 21 mm × 21 mm) holding 68 black platinum electrodes with a dimension of 40 μm × 40 μm, an inter-electrode distance of 200 μm holding an impedance of 10 kΩ at 1 kHz and a 5 μm thick SU-8 polymer layer for insulation were used. For culturing of the cells, each MEA was placed into a 35 mm culture dish (TPP, Trasadingen, Switzerland) holding 2 ml of DMNK^+^ medium.

Further, MEAs from MultiChannel Systems (MultiChannel Systems GmbH, Reutlingen, Germany) composed of 60 Titanium nitride electrodes with a diameter of 30 μm (59 recording electrodes, 1 internal reference electrode), an inter-electrode distance of 200 μm holding an impedance of <100 kΩ and a silicon nitride isolation were used. Further they hold a 6 mm high glass ring around the electrodes allowing the addition of 1 ml of DMNK^+^ medium.

### Electrophysiological Recordings

Spontaneous neuronal activity was measured by detecting extracellular voltage transients, induced by current flow through membranes of neurons. MEAs were incorporated into a Plexiglas chamber and mounted on an inverted microscope. To maintain the pH at 7.4 the cultures were superfused with an extracellular solution consisting of 145 mM NaCl, 4 mM KCl, 1 mM MgCl_2_, 2 mM CaCl_2_, 5 mM Hepes, 2 mM Na-pyruvate and 5 mM glucose, (all Sigma-Aldrich, St. Louis, MO, USA). The 68 electrodes were AC-coupled to an individual custom-made amplifier and data were digitized at a rate of 6 kHz with 12 bit resolution and stored on a hard disk for offline analysis as described previously (Tscherter et al., 2001). To control the A/D card (NI-DAQ-card, AT-MIO-64E-3, National Instruments, Ennetbaden, Switzerland) a custom-made LabVIEW software (National Instruments, Ennetbaden, Switzerland) was used. The contribution of inhibitory neurons in our cultures was analyzed under the treatment of a GABA A (10 μM gabazine) and glycine (1 μM strychnine, both Sigma-Aldrich, St. Louis, MO, USA) receptor antagonist and signals were recorded for 10 min (*n* = 7, from three independent experiments) (**Figure 3C**). To discriminate intrinsic neuronal activity and synaptic activity, pharmacological identification of postsynaptic responses was performed by application of a mixture of GABA A (10 μM gabazine), glycine (1 μM strychnine), α-amino-3-hydroxy-5-methyl-4-isoxazolepropionic acid/kainate (10 μM, CNQX) and *N*-methyl-D-aspartate (50 μM APV) receptor antagonists (all from Sigma–Aldrich, St. Louis, MO, USA) thus inhibiting all synaptic transmission (**Figures 3A,B**). This step was conducted at the end of every recording with each culture that showed a remaining burst activity. Further, to distinguish background noise from neuronal activity, every culture was treated as a final step with 1 μM TTX (Alomone Labs, Jerusalem, Israel), a potent voltage-gated sodium channel blocker, and recorded for 10 min. The resulting activity was then set as a zero reference.

For long-term time-dependent analysis measurements a commercially available MEA system (MultiChannel Systems GmbH, Reutlingen, Germany) was used to measure the activity 21 days (±2 days) after plating the cultures. This setup enabled to conduct measurements at different time points without the risk of contamination. The signals from the MEAs were amplified with a MEA2100 headstage and data were digitized with a 60-channel A/D converter at a rate of 25 kHz with 16 bit resolution. The measurement was controlled via MC_Rack software (MultiChannel Systems GmbH, Reutlingen, Germany) and the data were stored on a hard disk for further analysis. The temperature was controlled with an external heater unit (TC02, MultiChannel Systems GmbH, Reutlingen, Germany) set to 37.6°C. To eliminate evaporation and contamination during the experiment the MEAs were sealed with a semi-permeable membrane (ALA MEA-MEM, ALA Scientific Instruments, Westbury, NY, USA), (Potter and DeMarse, 2001). All recordings were conducted in DMNK^+^ medium. To discriminate intrinsic neuronal activity and synaptic activity, pharmacological identification of postsynaptic responses was performed as described above.

### Analysis of Spontaneous Activity

Event detection and further analysis was performed offline using IGOR (WaveMetrics, Inc., Lake Oswego, OR, USA) as described previously (Tscherter et al., 2001). The detected signals are fast voltage transients (<4 ms) corresponding to single action potentials or spikes in neuronal somata or axons (single unit activity) and are represented by single time markers called events. These are shown in event raster plots for each electrode (**Figure 2A**). Usually, they appeared in clusters (multi-unit activity) originating from closely timed action potentials of several neurons seen by one or multiple electrodes. These multi-unit activities were defined as burst (**Figure 2C**). Detected bursts were subsequently defined offline in IGOR by reaching a certain threshold set individually for each culture with the beginning being defined as the first event in a time window of at least 5 ms and the burst end being the last event in a time window of at least 10 ms. Network activity plots (**Figure 2B**) show the total activity of active electrodes within a sliding window of 10 ms shifted by a 1 ms step. For calculation of the spike rate (spikes/s per active electrode), spontaneously active electrodes were defined as electrodes showing a minimum of 0.1 detected events per second. Electrodes which detected fewer or 0 signals were not taken into account.

### BoNT Activity Assay

Due to the high toxicity of BoNTs, the handling requires appropriate safety measures. Solubilized neurotoxins were handled in a level 2 biosafety cabined equipped with high-efficiency particulate air filters. All consumables (e.g., pipette tips, falcon tubes) and medium that were exposed to the toxin, as well as the neurotoxin itself, were treated with 2 M NaOH (Sigma-Aldrich, St. Louis, MO, USA) and incubated for at least 1 h prior to discarding them in a separate container. These were autoclaved and disposed in the biological waste containers within the laboratory.

To assess the biologic activity of BoNT, 21 day (±2 days) old cultures were exposed to either 1.66, 16.6, or 166 pM of purified BoNT/A (A1 Hall Strain, Metabiologics, Madison, WI, USA) diluted in PBS and incubated at 37°C, 5% CO_2_ in 2 ml of DMNK^+^ medium. Cultures serving as control received the equivalent volume of PBS containing no toxin. After 6 h, cultures (treated and untreated) were washed twice with DMNK^+^ medium to remove all unbound toxin and neuronal activity was measured during 10 min as described beforehand in the electrophysiological recordings section. Serving as a negative control, cultures were treated with 166 pM heat inactivated BoNT/A (95°C, 5 min) in PBS and incubated for 6 h at 37°C, 5% CO_2_.

For long-term measurements 21 day (±2 days) old cultures were exposed to either 1.66 pM of purified BoNT/A (A1 Hall Strain, Metabiologics, Madison, WI, USA) or 33 Units/ml of onabotulinumtoxin A (Botox^®^, Allergan Inc., Dublin, Ireland) diluted in PBS and incubated at 37°C, 5% CO_2_ in 1 ml of DMNK^+^ medium. Neuronal activity measured in all cultures at the beginning of the experiment (0 h) served as baseline activity. Upon treatment with the toxin, the cultures were transferred after 6, 12, and 24 h from the incubator to the MultiChannel Systems electrical recording system and neuronal activity was measured. Before each recording the cultures were allowed to settle for 5 min before spontaneous neuronal activity was measured for 2 min. After completion of the recordings the cultures were transferred back to the incubator.

### Immunocytochemistry

On day 7 of differentiation, EBs were dissociated as described in Section “Mouse Embryonic Stem Cell Culture” and 2 × 10^5^ cells/ml in 500 μl DMNK^+^ medium supplemented with growth factors were plated on round cover glasses (Ø 12 mm, HUBERLAB, Aesch, Switzerland) which were previously coated for 30 min at 37°C with Matrigel^TM^ (Corning, Corning, NY, USA) diluted 1:10 in DMNK^+^ medium. The cells were then cultured at 37°C, 5% CO_2_ for up to 21 days, changing medium every 3 days. Following, cells were fixed in 4% paraformaldehyde (Sigma-Aldrich, St. Louis, MO, USA) in PBS for 10 min, washed three times with PBS and permeabilized with 0.1% Triton X-100 for 5 min (Sigma–Aldrich, St. Louis, MO, USA). Prior to staining, cells were blocked for 2 h with 2% bovine serum albumin and 0.01% Triton X-100 (both Sigma–Aldrich, St. Louis, MO, USA). Primary antibodies against β-III Tubulin (monoclonal mouse anti β-III Tubulin, 1:500, R&D Systems, Minneapolis, MN, USA; polyclonal rabbit anti β-III Tubulin, 1:500, Abcam, Cambridge, UK), synaptic vesicle glycoprotein isoform A/B/C (monoclonal mouse anti SV2, 1:100), GD1a ganglioside (monoclonal mouse anti GD1a, 1:10), GT1b/2b ganglioside (monoclonal mouse anti GT1b/2b, 1:10) (SV2 antibody developed by Buckley, K.M., GD1a, and GT1b/2b antibodies developed by Schnaar, R.L. obtained from the Developmental Studies Hybridoma Bank, created by the NICHD of the NIH and maintained at The University of Iowa, Department of Biology, Iowa City, IA, USA), synaptic vesicle glycoprotein isoform C (polyclonal rabbit anti SV2C, 1:500), SNAP-25 (monoclonal mouse anti SNAP-25, 1:500), synapsin 1a/b (monoclonal mouse anti Syn1, 1:500), VGLUT 2 (monoclonal mouse anti VGLUT2, 1:500), glutamate decarboxylase 1 (polyclonal rabbit anti GAD1, 1:500) (all Synaptic Systems, Goettingen, Germany), and GFAP (polyclonal rabbit anti GFAP, 1:500) (Chemicon, Merck Millipore, Darmstadt, Germany) were incubated over night at 4°C in PBS with 2% bovine serum albumin and 0.01% Triton X-100. The samples were washed three times with PBS and secondary antibodies conjugated with Alexa 488, Alexa 555, and Alexa 647 (all Invitrogen, Paisley, Scotland, UK) were incubated the following day for 2 h at room temperature diluted 1:500 in 2% bovine serum albumin and 0.01% Triton X-100. Cells were washed three times in PBS and mounted in Fluoroshield^TM^ with DAPI (Sigma–Aldrich, St. Louis, MO, USA) for nuclear staining. Images were acquired with a Zeiss laser scanning confocal microscope 710 using a Zeiss Plan-Apochromat 60x 1.4 NA objective and a Nikon Eclipse Ti microscope using Nikon-Plan Fluor 40x 1.0 NA and 63x 1.4 NA objectives.

### Statistical Analysis

Graph Pad Prism software (version 6.0) was used for statistical analyses. Differences between unpaired groups were evaluated either using the two-tailed unpaired Mann–Whitney test or the unpaired *t*-test. For comparison of paired groups a paired *t*-test was conducted. A *p*-value < 0.05 (two-tailed) was considered as statistically significant.

## Results

### Neuronal Cultures Derived from mESCs Form Synapses and Express the Proteins Necessary for BoNT/A Intoxication

**FIGURE 1:**
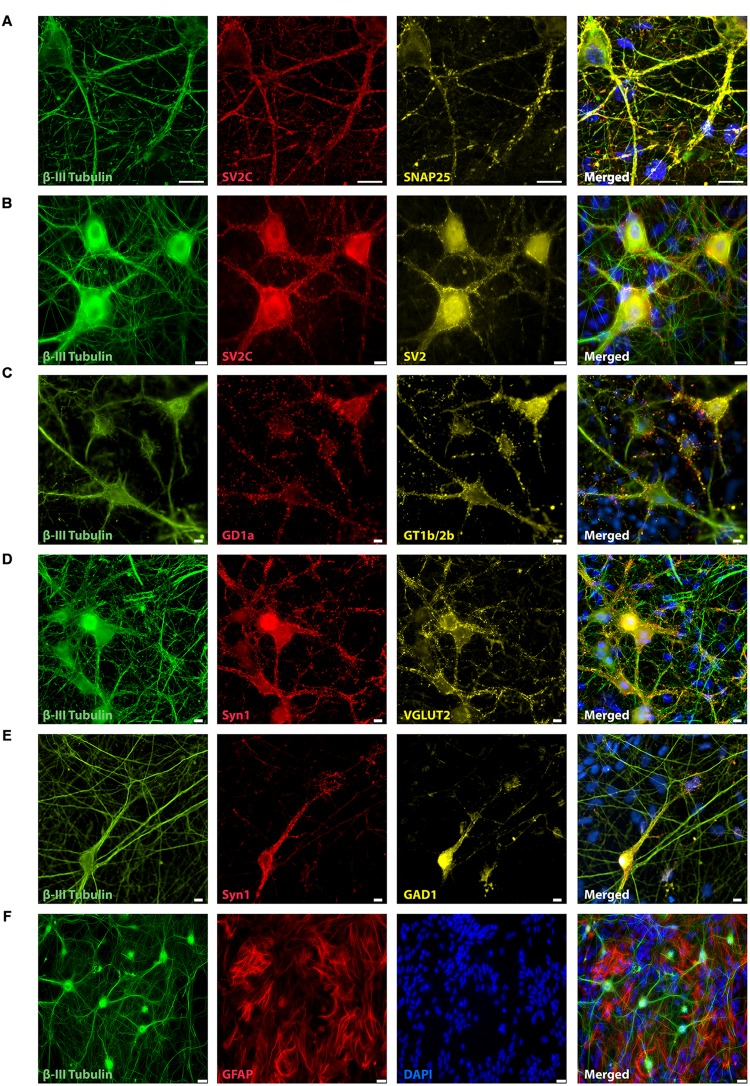
**Representative immunocytochemistry demonstrating the expression of neuronal, synaptic, glutamatergic, GABAergic, and glial markers in cultures 21 days after plating.** Neuronal cultures were immunostained against β-III Tubulin **(A–F)**; SNAP-25 **(A)**; SV2 isoform A–C **(A,B)**; polysialo gangliosides GD1a and GT1b/2b **(C)** Syn1 **(D,E)**; glutamatergic neurons (VGLUT2, **D**); GABAergic neurons (GAD1, **E**) and glial cells (GFAP, **F**). Shown also are DAPI nuclear staining and the merged images. Scale bar is 10 μm **(A–E)**, respectively, 25 μm for the bottom panel **(F)**.

**FIGURE 2:**
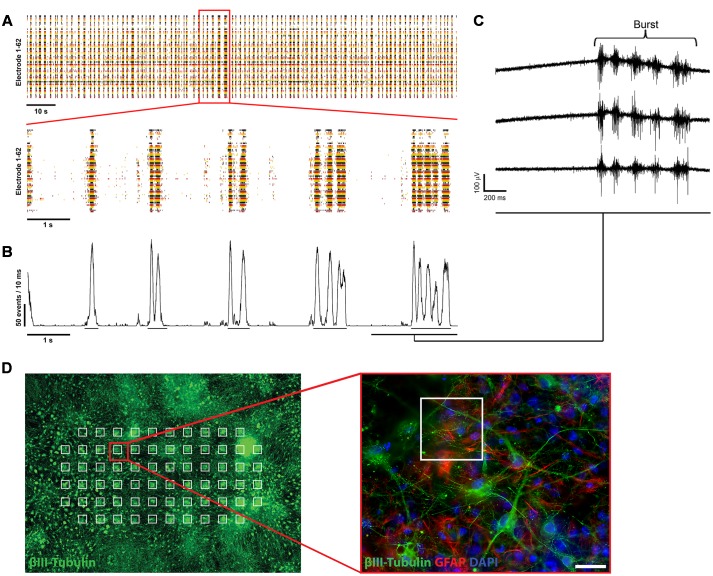
**Electrophysiological characterization of mESN cultured on MEAs.** (**A**, top) Raster plot of 62 single electrodes (represented by different colored lines) showing high spontaneous neuronal activity of a culture 21 days after plating. The formation of bursts is based on spontaneous intrinsic activity and recurrent excitation of the neuronal network through synaptic transmission. Events represent the neuronal activity recognized by the detector (see Materials and Methods). (**A**, bottom) 10 s excerpt of the raster plot showing individual bursts. **(B)** Network activity plot. This plot visualizes the amount of activity in the whole neuronal culture network during the time illustrated in the raster plot shown in **(A)**. It shows the number of events detected from all active electrodes with a sliding window of 10 ms shifted by a 1 ms step. Visible by the underlines are the beginning and ending of detected oscillating burst activities. **(C)** Raw data traces from three single MEA electrodes. **(D)** Schematic representation of mESN cultured for 21 days on a MEA immunostained for β-III Tubulin (green), GFAP (red), and DAPI (blue). Highlighted in white are the positions of each electrode. Scale bar is 20 μm.

Three days after plating (data not shown) and throughout the next 21 days, mESN expressed the post-mitotic neuronal marker β-III Tubulin (**Figures 1A–F**, **2D**). Immunocytochemistry performed 21 days after plating the cells revealed, that a large proportion of post-mitotic neurons expressed the VGLUT 2, a known marker for glutamatergic neurons (**Figure 1D**). However, only a minority of these cells were immunoreactive for GAD1, a marker for GABAergic neurons (**Figure 1E**). Co-staining with the universal pre-synaptic neuronal marker Syn1 proved the presence of synaptic vesicles in axons of β-III Tubulin positive cells (**Figures 1D,E**). In addition, neurons expressed the polysialo gangliosides GD1a and GT1b/2b, both known to mediate the initial anchorage of BoNT/A toward the presynaptic membrane (**Figure 1C**). Furthermore, the expression of all three pre-synaptic membrane receptor isoforms SV2 C/A/B (order expresses decreasing neurotoxin affinity) (Mahrhold et al., 2006; Rummel, 2013) (**Figures 1A,B**), necessary for docking and cytosolic uptake of BoNT/A as well as its target protein SNAP-25 (**Figure 1A**) further confirmed that these cells expressed the necessary components used by BoNT to inhibit synaptic activity. After 21 days in culture, a high amount of GFAP were observed, a proteins expressed by numerous cell types of the central nervous system including astrocytes (**Figures 1F**, **2D**).

### mESN Show High Spontaneous Synaptic Activity

First single spikes, recorded from individual electrodes were measured beginning 3 days after plating of the cells. At 6 days after plating the cultures showed first synchronous spontaneous activity over several electrodes simultaneously and reaching a plateau at 21 days after plating, represented in the formation of oscillating bursts (**Figure 2A**). The size of the bursts was random and did not have any obvious periodicity and their occurrence could not be predicted.

**FIGURE 3:**
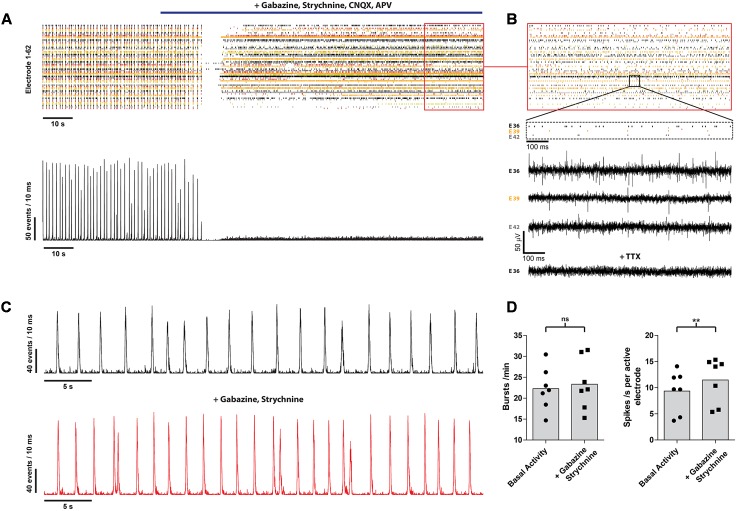
**mESN cultured on MEAs for 21 days show functional glutamatergic and GABAergic synapses with network characteristics.** **(A)** Event raster plot and corresponding network activity plot of activity recorded following the addition of gabazine, strychnine, APV, and CNQX (highlighted by the blue bar). This combination of receptor antagonists leads to an inhibition of synaptic transmission and thus preventing the formation of bursts as well as resulting in a dramatic decrease of the average spike rate. **(B)** Note that under these conditions asynchronous intrinsic activity is still visible on several electrodes. Raw data traces are shown for three electrodes (E 36, E 39, E 42) as well as for one electrode (E 36) after application of 1 μM TTX. **(C)** Network activity plot showing the activity prior (top) and after treatment with 10 μM gabazine and 1 μM strychnine. **(D)** Note the slight increase in burst (left) as well as average spike rate (right). Data are given as mean; paired *t*-test, two tailed; ^∗∗^*P* < 0.01; ns signifies as not significant.

Next, we analyzed the contribution of the different neurotransmitters to spontaneous postsynaptic events by pharmacological interference. Inhibition of GABA A, glycine, α-amino-3-hydroxy-5-methyl-4-isoxazolepropionic acid/kainate and *N*-methyl-D-aspartate receptors completely abolished the remaining occurrence of bursts in every analyzed culture (*n* = 60) (**Figure 3A**). However, asynchronous intrinsic activity was still detectable, indicating that the neurons were still able to fire single action potentials (**Figure 3B**). Inhibition of the GABA A and glycine receptor by gabazine and strychnine, thus blocking the effects of inhibitory neurons, led to a slight increase of the burst rate (22.8 ± 5.11 vs. 23.4 ± 6.1 bursts/min; *n* = 7; from three independent experiments), as well as mean spike rate (9.4 ± 3.9 vs. 11.5 ± 4.3 spikes/s per active electrode; *p* < 0.01; *n* = 7; from three independent experiments) (**Figures 3C,D**). This demonstrates the presence of inhibitory synaptic connections involving GABA A and glycine receptors, in accordance with our immunocytochemistry findings on the presence of a small amount of GABAergic neurons (**Figure 1D**). Finally, all spontaneous activity was eliminated by applying 1 μM TTX, indicating that the detected voltage transients originated from neuronal activity (**Figure 3B**, bottom raw data trace).

Collectively, these data demonstrate the functional development of glutamatergic and GABAergic synapses within our mESC-derived neuronal network.

### mESN Cultured on MEAs Can Sensitively Detect the Biological Activity of BoNT/A in a Dose- and Time-Dependent Manner

**FIGURE 4:**
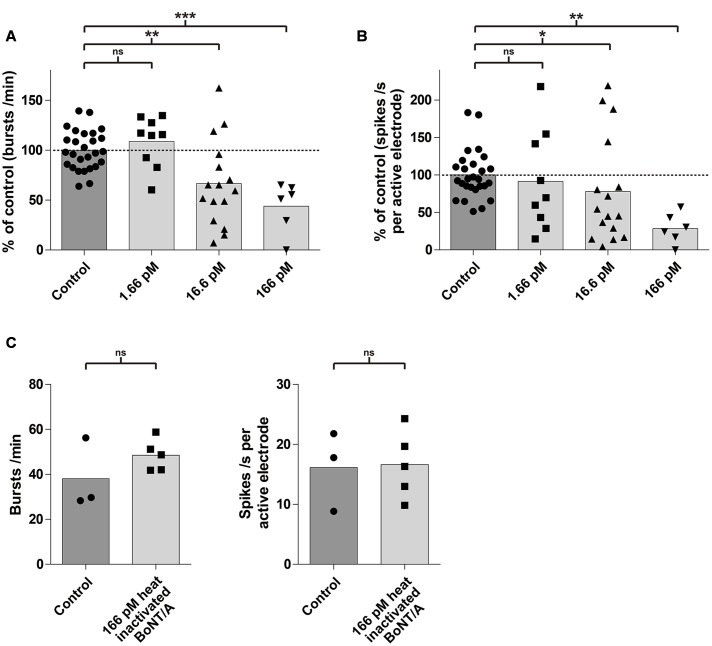
**mESN grown on MEAs are susceptible to intoxication by BoNT/A in a dose-dependent manner.** Treatment and incubation for 6 h with 16.6 and 166 pM BoNT/A resulted in a significant decrease of the burst rate and the mean spike rate. Cultures receiving no toxin served as control. **(A)** Reduction of the burst rate compared to the control group. **(B)** Reduction of the mean spike rate compared to the control group. Mann–Whitney two tailed test; ^∗^*P* < 0.05; ^∗∗^*P* < 0.01; ^∗∗∗^*P* < 0.001; ns signifies as not significant. **(C)** Treatment and incubation with 166 pM heat inactivated BoNT/A showed no significant difference in burst (left) as well as average spike rate (right). Paired *t*-test, two tailed; ns signifies as not significant.

Next, we evaluated the effect of BoNT/A exposure on the average network activity, defined by changes in burst and spike rates, in a dose- and time-dependent fashion. Exposure to BoNT/A for 6 h resulted in a significant decrease of the burst rate for toxin concentrations of 166 pM (43.9 ± 24.9%; *p* < 0.0005; *n* = 6) and 16.6 pM (66.7 ± 42.5%; *p* < 0.005; *n* = 16) compared to untreated cultures (100 ± 19.7%; *n* = 27) (**Figure 4A**). A significant decrease in the mean spike rate was observed for toxin concentrations of 166 pM (28.7 ± 20.0%; *p* < 0.005; *n* = 6) and 16.6 pM (78.0 ± 70.9%; *p* < 0.05; *n* = 16) compared to untreated cultures (100 ± 31.9%; *n* = 27) (**Figure 4B**). However, no significant decrease in burst rate (108.7 ± 25.2%; *p* = 0.25; *n* = 9) (**Figure 4A**) and mean spike rate (91.5 ± 67.3%; *p* = 0.38; *n* = 9) (**Figure 4B**) was observed upon treatment with 1.66 pM BoNT/A for 6 h.

To exclude that the observed effect of the toxin on neurons may be due to protein properties unrelated to its biological activity, cultures (*n* = 5) were treated with 166 pM of heat inactivated (5 min, 95°C) BoNT/A for 6 h. It has previously been shown, that heating BoNT/A for 15 s at 72°C was sufficient to reduce its toxic activity by more than 99.99% (Weingart et al., 2010). Compared to a control (untreated group, *n* = 3), we could not detect any significant difference in the burst or spike rate after 6 h (**Figure 4C**) indicating that the decrease of activity was indeed caused by the specific biological activity of BoNT/A.

**FIGURE 5:**
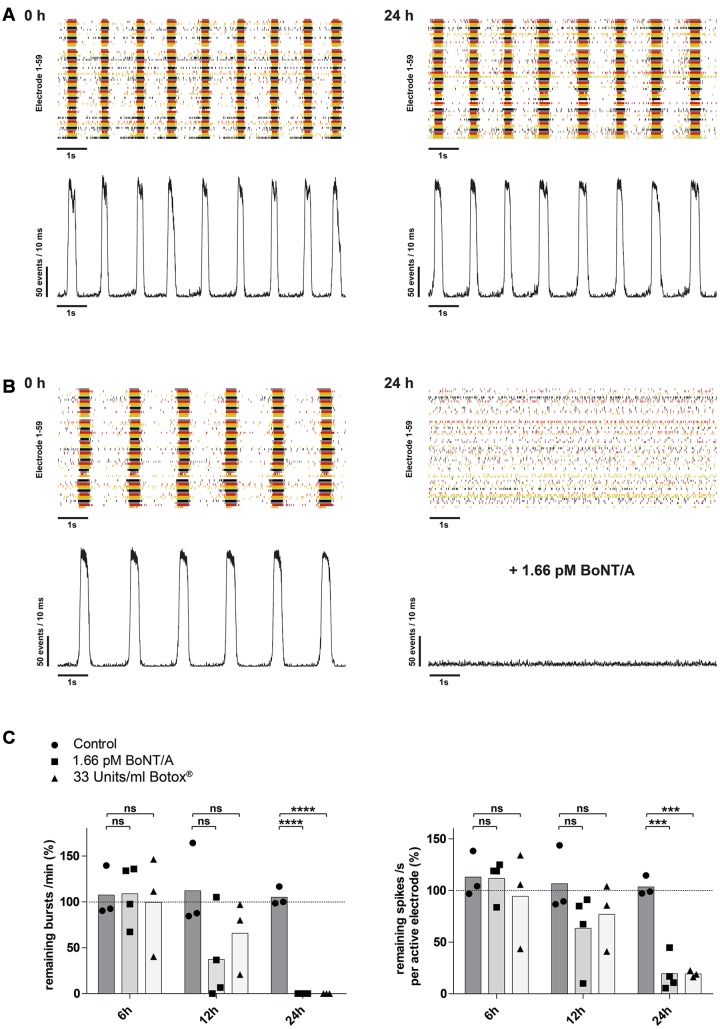
**Time-dependent reduction of synaptic transmission upon addition of BoNT/A holotoxin and complex.** **(A)** Raster plot and corresponding network activity plot showing spontaneous burst activity of a 21-day-old control culture prior (left) and 24 h after receiving 10 μl of PBS (right). **(B)** Upon treatment and incubation with 1.66 pM BoNT/A for 24 h a complete loss of synchronous burst activity is visible (right). Seen on the left is the same culture prior to addition of the toxin serving as the base activity at 0 h (left). **(C)** Decrease of the burst rate (left) and the mean spike rate (right) upon treatment and incubation with 1.66 pM BoNT/A or 33 Units/ml Botox^®^ at the indicated time points. Each culture was normalized toward its base activity measured at time point 0 h (highlighted by the dotted line). The reduction of the activity was then compared toward untreated cultures serving as a control. Unpaired *t*-test, two tailed; ^∗∗∗^*P* < 0.001; ^∗∗∗∗^*P* < 0.0001; ns signifies as not significant.

It has been shown, that BoNT/A leads to a time-dependent increase of SNAP-25 cleavage as well as decrease in the network activity (Pellett et al., 2011; Beske et al., 2015). We therefore examined whether longer exposure periods with 1.66 pM BoNT/A would lead to a decrease of the network activity. In control cultures exposed to PBS only, there was no significant changes over time in either the burst or mean spike rate (**Figures 5A,C**). After 12 h of incubation with 1.66 pM BoNT/A the overall burst rate of the neuronal cultures was reduced by 62.9% (37.1 ± 48.0%; *n* = 4) and the mean spike rate was reduced by 36.6% (63.4 ± 36.9%; *n* = 4) comparing to the baseline activity (highlighted by the dotted line) determined prior to treatment (**Figure 5C**). At 24 h post treatment with 1.66 pM BoNT/A a complete loss of synaptical driven activity was observed resulting in the complete absence of burst formation when compared to the baseline activity (**Figure 5B**, right panel and **Figure 5C**, left panel). Further, the average spike rate was reduced by 80.5% (19.5 ± 17.4%; *n* = 4) when compared to the baseline activity. Solely the asynchronous intrinsic activity was detectable (**Figure 5B**, right panel and **Figure 5C**, right panel). By comparing the untreated control group receiving only PBS toward BoNT/A treated cultures 24 h post treatment, significant decreases of both the burst rate (105.0 ± 10.1%; *n* = 3) vs. (0.0 ± 0.0%; *p* < 0.0001; *n* = 4) (**Figure 5C**, left panel) and the mean spike rate (103.6 ± 9.5%; *n* = 3) vs. (19.5 ± 17.4%; *p* < 0.001; *n* = 4) (**Figure 5C**, right panel) were observed.

Pharmaceutical preparations with onabotulinumtoxin A (Botox^®^, Botox Cosmetics^®^, Vistabel^®^, Vistabex^®^) contain the 150 kDa holotoxin BoNT/A and several non-toxic clostridial accessory proteins which are covalently bonded to the 150 kDa holotoxin forming a BoNT/A complex. We therefore assessed, if BoNT/A complex interferes with the sensitivity or kinetics of the described assay. Upon treatment with 33 Units/ml of Botox^®^, corresponding to approximately 1.66 pM of BoNT/A based on its reported specific activity, and incubation for 24 h a complete loss of synaptical driven activity was observed resulting in the complete absence of burst formation when compared to the baseline activity (**Figure 5C**, left panel). Comparing the average spike rate 24 h post treatment with an untreated control group, a significant decrease in activity was observed (103.6 ± 9.5%; *n* = 3) vs. (19.2 ± 3.2%; *p* < 0.001; *n* = 3) (**Figure 5C**, right panel).

Overall these data indicate, that mESN grown on MEAs can detect the biological activity of BoNT/A holotoxin in a time- and dose-dependent manner resulting in a decrease of synaptically mediated neuronal activity. Further, no impairment regarding kinetics or sensitivity was observed using BoNT/A complex that is contained in the pharmaceutical preparation Botox^®^.

## Discussion

The *in vivo* MBA was introduced in the 1920s and is still considered as the gold standard method for the detection of BoNTs. It has a high sensitivity, with detection limits at 20 pg/ml (0.13 pM BoNT/A), can detect all steps of BoNT intoxication and is able to detect all functionally active BoNT serotypes (Lindstrom and Korkeala, 2006; Dorner et al., 2013). However, due to considerable ethical concerns and a number of disadvantages there is a high demand for the replacement of the MBA. Here, neuronal cell-based assays can provide an alternative method for the detection of BoNT activity in pharmaceutical preparations. BoNT/A leads to cleavage of the essential SNARE protein SNAP-25 thus leading to the inhibition of neurotransmitter release from the pre-synaptic compartment in peripheral neurons (Rossetto et al., 2014). Therefore, treatment of *in vitro* cultures should lead in theory to a reduction or elimination of synaptical driven network responses.

In this study, we demonstrated that mESN grown on MEAs can serve as a physiologically relevant, cell-based model for the detection of BoNT/A holotoxin and complex. We showed that mESN form functional networks, express the receptors and proteins necessary for BoNT/A intoxication and show high spontaneous activity resulting in the formation of bursts (**Figure 2A**). Further, upon treatment for 24 h with 1.66 pM BoNT/A or 33 Units/ml Botox^®^ a complete silencing of synaptic transmission was observed and this decrease in activity is a function of concentration and time (**Figures 4A,B**, **5C**). This observation is consistent with other published data where the incubation of neuronal cultures with 20 pM of BoNT/A for 20 h led to a cleavage of 95% of total cellular SNAP-25 leading to an impairment of synaptic activity (McNutt et al., 2011; Beske et al., 2015). In addition, the persistence of asynchronous intrinsic activity after BoNT/A treatment indicates that the effect is not attributable to overall cytotoxicity (**Figure 5B**, right panel). The observed sensitivity towards BoNT/A is in agreement with other published studies which showed sensitivity in the range of 0.03–500 pM. The majority of these studies, however, rely on the detection of SNARE cleavage in cell lysates by Western blot (McNutt et al., 2011; Pellett et al., 2011; Fernandez-Salas et al., 2012; Whitemarsh et al., 2012), or by direct/ capture ELISA (Nuss et al., 2010). Although high sensitivity can be reached by cleavage assays, most approaches require cell lysis and therefore exclude longitudinal data sampling. Kiris et al. (2011) showed in a previous study a possible high-throughput method by detecting SNARE cleavage upon BoNT/A treatment in live cells by quantitative immunofluorescence allowing for continuous data sampling. However, only concentrations down to 25 pM BoNT/A were tested (Kiris et al., 2011). An interesting approach was conducted by Dong et al. (2004) where a SNARE-FRET construct was transiently expressed in PC12 cells which allowed the study of toxin substrate cleavage *in situ.* Though they could successfully detect BoNT/A activity, the assay lacked the required sensitivity (50 nM) and incubation times of 96 h were necessary (Dong et al., 2004). On the contrary, using electrophysiological methods and observing the inhibition of synaptic transmission in response to BoNT poses a non-invasive and sensitive method allowing for continuous recording. Two previous studies reported the use of primary cortical and spinal neurons grown on MEAs as a detection model for BoNT/A. They showed a sensitivity toward BoNT/A of 13 pM after 24 h (Pancrazio et al., 2014) and 1.3 pM after 48 h (Scarlatos et al., 2008). For both studies either E16-17 mice embryos (Pancrazio et al., 2014) or E17-18 rat embryos (Scarlatos et al., 2008) were sacrificed and primary neurons harvested. By using whole-cell patch-clamp recordings on mESN a recently published study showed a calculated half maximal inhibitory concentration value of 55 fM for BoNT/A (Beske et al., 2016). Although they were able to show sensitivity equal to the MBA, patch-clamp electrophysiology is a low-throughput method requiring specialized techniques and trained staff. In contrast, commercially available MEA systems allow for a high-throughput screening. This has been shown in different studies assessing drug effects on cardiomyocytes derived from human embryonic stem cells or human-induced pluripotent stem cells (Mandenius and Meyer, 2013; Clements and Thomas, 2014; Gilchrist et al., 2015; Clements, 2016). In addition, they offer tailored software packages that allow non-trained staff to operate the system. However, differentiation of mESCs toward neurons still needs trained staff and during the first week of differentiation hands-on time is required. Here, the usage of commercially available cryopreserved human induced pluripotent stem cell-derived neurons could reduce hands-on time to a minimum and only basic knowledge of tissue culture techniques is required. These commercially available cells undergo strict quality controls, thus enabling reproducible neuronal cultures. Further, it has been shown that human induced pluripotent stem cell-derived neurons are highly sensitive to BoNT/A with a half maximal effective concentration of around 0.3 mouse LD_50_ Units (Whitemarsh et al., 2012). A main drawback, however, is the low sensitivity toward BoNT/B being around 16 mouse LD_50_ Units. It is believed that this is due to a single point mutation in the human neuronal protein receptor synaptotagmin II which decreases the affinity toward BoNT/B (Strotmeier et al., 2012; Rummel, 2013). Therefore, one must considerer species specificity when detecting different BoNT serotypes.

The formation of spontaneous activity observed in our cultures is a common characteristic during development of neuronal networks both *in vivo* and *in vitro* and our data is comparable to other studies showing over time the formation of spontaneous burst activity in primary networks *in vitro* (Maeda et al., 1995; van Pelt et al., 2004; Czarnecki et al., 2012) and mESC-derived networks *in vitro* (Ban et al., 2007). These bursts are based on spontaneous intrinsic activity and recurrent excitation of the neuronal network through synaptic transmission (Darbon et al., 2002; Yvon et al., 2007). Our cultures are composed of a heterogeneous mixture of excitatory and inhibitory neurons which is comparable to previous studies that show the presence of GABAergic and glutamatergic neurons in mESC-derived cultures (Ban et al., 2007; Beske et al., 2015) or cortical cultures (Czarnecki et al., 2012). Clostridial toxins affect not only cholinergic peripheral synapses but also possess the ability to affect all kind of synapses (Verderio et al., 2007; Akaike et al., 2010; Beske et al., 2015). Interestingly, it has been observed *in vitro* that spinal cord synapses as well as synapses of mESN differ in their susceptibility toward BoNTs where GABAergic neurons show a higher sensitivity than glutamatergic neurons (Akaike et al., 2010; Beske et al., 2015). It is postulated that this effect is due to the higher firing rate of inhibitory neurons leading to a higher rate of synaptic vesicle endocytosis and thus causing a more rapid uptake of the toxin (Beske et al., 2015). These synapse-specific differences in latency would therefore lead to a biphasic response where initially an increase of network activity would be observed. This effect has been reported by different studies *in vitro* where primary cortical neurons (Scarlatos et al., 2008), spinal cord neurons (Pancrazio et al., 2014) or mESN (Beske et al., 2015) show a synapse subtype specific latency toward intoxication leading to an initial increase of network activity. In the present study, upon treatment with BoNT/A we did not observe an initial increase of network activity. Previous studies (Pancrazio et al., 2014; Beske et al., 2015) showed that this initial increase occurs as early as 30 – 60 min after BoNT/A addition and is therefore not detectable in the present experimental setup. The treatment with GABA A and glycine receptor antagonists, thus isolating the effects of inhibitory neurons, showed only a minor increase in the burst as well in the total network activity (**Figure 3D**). Comparing these results with our immunocytochemistry characterization and previously published studies, this data suggests that only a small population of neurons show a GABAergic phenotype. To increase the sensitivity of our *in vitro* bioassay and achieve a shorter readout time one could consider using a differentiation protocol yielding a higher GABAergic neuronal population (Westmoreland et al., 2001; Chatzi et al., 2009). In addition, the high amount of glial cells in our cultures reflects the prolonged period of *in vitro* differentiation which is required for a network formation (**Figure 1F**). An increased number of GFAP positive cells over time is concordant with *in vivo* developmental progression as well as with neural stem cell differentiation studies *in vitro* (Sauvageot and Stiles, 2002; Abranches et al., 2009).

In summary, we have shown that electrophysiological detection of network activity provides a physiological relevant readout of BoNT/A intoxication. Further, we could show that BoNT/A complex and the presence of pharmaceutical excipients do not alter the sensitivity or kinetics of the described assay. Unlike most cell-based assays which are customized for specific BoNT serotypes, mESN can be used for detection of a range of BoNT serotypes (Beske et al., 2016). Further, culturing mESN on MEAs allows for a non-invasive and continuous monitoring of synaptic transmission reflected by burst activity and unlike primary cultures no animals are required for the assay. In addition, the problem of scalability regarding primary neuron harvest is not an issue and the used MEA setup allows for a prospective high-throughput screening. However, a limitation at the time being is the restricted sensitivity to concentrations down to 1.66 pM. Future studies will be necessary to better understand the effects of BoNTs on neuronal networks and increase the sensitivity. Once these issues are addressed, the present system holds the potential to reduce animal use for BoNT detection and activity determination.

## Author Contributions

SJ conceived the study design, performed the experiments, collected and analyzed the data, and wrote the manuscript. DG contributed to the study design and the data analysis and revised the manuscript. MH collected data. AT collected data and revised the manuscript. M-AA revised the manuscript. SL initiated and supervised the project, contributed to the study design and the data analysis and revised the manuscript.

## Conflict of Interest Statement

The authors declare that the research was conducted in the absence of any commercial or financial relationships that could be construed as a potential conflict of interest.

## Abbreviations

APV: (*2R*)-amino-5-phosphonovaleric acid
BoNT: botulinum neurotoxin
CNQX: 6-cyano-7-nitroquinoxaline-2,3-dione
DAPI: 4’,6-Diamidin-2-phenylindol
EB: embryoid body
ELISA: enzyme-linked immunosorbent assay
FRET: Förster resonance energy transfer
GABA A: ɣ-aminobutyric acid A
GAD1: glutamate-decarboxylase 1
GFAP: glial fibrillary acidic protein
HC: heavy chain
LC: light chain
LD_50_: median lethal dose
MBA: mouse bioassay
MEA: multi-electrode array
mESC: mouse embryonic stem cell
mESN: mouse embryonic stem cell-derived neurons
PBS: phosphate-buffered saline
SNARE: soluble *N*-ethyl maleimide sensitive factor attachment protein receptor
SV2: synaptic vesicle glycoprotein 2
Syn 1: synapsin 1a/b
SNAP-25: synaptosomal-associated protein of 25 kDa
TTX: tetrodotoxin
VGLUT 2: vesicular glutamate transporter 2
